# Measurement of Micro Burr and Slot Widths through Image Processing: Comparison of Manual and Automated Measurements in Micro-Milling

**DOI:** 10.3390/s21134432

**Published:** 2021-06-28

**Authors:** Fatih Akkoyun, Ali Ercetin, Kubilay Aslantas, Danil Yurievich Pimenov, Khaled Giasin, Avinash Lakshmikanthan, Muhammad Aamir

**Affiliations:** 1 Department of Mechanical Engineering, Faculty of Engineering, Adnan Menderes University, Aydın 09010, Turkey; fatih.akkoyun@adu.edu.tr; 2 Department of Mechanical Engineering, Faculty of Engineering and Architecture, Bingol University, Bingol 12000, Turkey; 3 Department of Mechanical Engineering, Faculty of Technology, Afyon Kocatepe University, Afyonkarahisar 03200, Turkey; aslantas@aku.edu.tr; 4 Department of Automated Mechanical Engineering, South Ural State University, Lenin Prosp. 76, 454080 Chelyabinsk, Russia; danil_u@rambler.ru; 5 School of Mechanical and Design Engineering, University of Portsmouth, Portsmouth PO1 3DJ, UK; khaled.giasin@port.ac.uk; 6 Department of Mechanical Engineering, Nitte Meenakshi Institute of Technology, Bengaluru 560064, India; avinash.laks01@gmail.com; 7 School of Engineering, Edith Cowan University, Joondalup, WA 6027, Australia; m.aamir@ecu.edu.au

**Keywords:** computer vision, image processing, micro-machining, slot and burrs, measurement, characterization

## Abstract

In this study, the burr and slot widths formed after the micro-milling process of Inconel 718 alloy were investigated using a rapid and accurate image processing method. The measurements were obtained using a user-defined subroutine for image processing. To determine the accuracy of the developed imaging process technique, the automated measurement results were compared against results measured using a manual measurement method. For the cutting experiments, Inconel 718 alloy was machined using several cutting tools with different geometry, such as the helix angle, axial rake angle, and number of cutting edges. The images of the burr and slots were captured using a scanning electron microscope (SEM). The captured images were processed with computer vision software, which was written in C++ programming language and open-sourced computer library (Open CV). According to the results, it was determined that there is a good correlation between automated and manual measurements of slot and burr widths. The accuracy of the proposed method is above 91%, 98%, and 99% for up milling, down milling, and slot measurements, respectively. The conducted study offers a user-friendly, fast, and accurate solution using computer vision (CV) technology by requiring only one SEM image as input to characterize slot and burr formation.

## 1. Introduction

Workpiece optimization is an important procedure in micro-machining applications [1,2,3,4]. Today, micro-machining is widely used in the manufacturing of micro-sized parts for various applications, such as electronics, optics, automotive, aerospace, and biomedical [5,6,7,8]. Despite the high performance of the micro-machining process, the quality of micro-sized parts is related to the burr formation [5,6,9,10]. Especially in studies on micro-machining processes and working piece analyses, the precision measurement on slot and burr widths is necessary for better investigation of the micro-machining process and optimization of the machining conditions [11,12,13,14]. In the micro-machining process, cutting conditions and cutting parameters significantly affect the burr formation [15,16,17,18]. To minimize burr formation, new cutting conditions are determined by measuring burr dimensions after the micro-machining process. Before the micro-machining process, finite element modeling or mathematical modeling based on response surface methodology (RSM) are applied to decrease burr size [19,20,21,22,23,24]. Considering the complex geometrical shapes of the slot and burr on a machined workpiece, accurate, rapid, and practical measurement of slot and burr parameters using SEM images is a worthwhile utility for fundamental research [5,25,26,27]. Some characteristics make IN−718 difficult to cut, such as high strength, high degree of work hardening, poor thermal conductivity, and the high tendency of a built-up edge. Burr formation changes significantly due to these characterizations. The burr formation and surface quality need to be examined quickly by new methods [28].

Various methods are often used by researchers for slot and burr measurements [2,15,19,29,30]. One of them is to use a screen caliper to measure the slot and burr widths of an image that is captured using SEM [31,32]. In this approximation, an SEM image measured with a screen caliper using pixel information is used to determine the parameters of the working piece, which highly depends on the user-related error [32,33,34]. This method is inconvenient and manual measurement is required for each image, which is a long time process for investigating a large number of SEM images [31,32]. The next procedure for slot and burr widths measurement is to use a three-dimensional optical profilometer, which is commercially available from technology labs or various companies [11,35,36]. Although the profilometer instruments make the measurement very practical and precise, these are mostly present in well-funded labs because of their relatively higher costs. In addition, it is necessary to wait a few minutes for the result for each sample [31,32]. An artificial neural network (ANN) is another method to predict the formation of burrs in micro-drilling [37,38]. Ahn et al. [37] conducted a study to predict the burr formation in micro-drilling of ductile metals, which was found to have around an 80% accuracy. The other approach is to use image processing software to measure the slot and burr widths of micro-milling [5,39,40]. Tuiran et al. [39] analyzed burrs formed in micro-milling using image processing. An averaged 84.46% accuracy was found between spindle speed and burr formation. The image processing technology is a well-known technique with high accuracy and the ability to measure two-dimensional distances with different properties expressed mathematically for a large number of images. However, due to the license costs of the advanced image processing software, such as Neural Wear, and the lack of pinpoint solutions, these are not available in many research facilities [41,42]. There are also few open-sourced approaches for measuring slot borders and burr lengths on a machined workpiece using computer vision technology for example, ImageJ, which was released by the National Institute of Health of the United States of America [43]. It is published as free software that can be used to measure the slot and burrs widths from a captured image. Nevertheless, the image processing tools are limited and not proper for situations that may need multiple threshold operations for measuring complex SEM output in the same image. In laboratories with limited resources, the screen caliper method is one of the widely used methods for investigating slot and burr parameters [31,32]. Using various optical systems, burrs can be determined, and their dimensions can be measured. Camera systems, SEM, confocal microscopy, and laser triangulation are among the most important optical systems [44]. Methods, such as the triangulation method, the conoscopic holography method, and the interferometry method, have been developed for the measurement of micro burr geometry in the micro-drilling process [45]. A measurement system based on a contour detection algorithm has been designed and aims to detect burrs and environmental defects in cast parts. However, the machine vision system developed was limited to macro dimensional burr measurements [5]. Medeossi et al. [5] found 87% for the maximum accuracy of burr width evaluation in micro-milling. A laser-based measuring system is designed for the characterization and control of micro burr parameters [46]. Despite all these developments, obtaining measurement results is not fast enough. Therefore, there is a requirement for an open-sourced, user-friendly, high speed, automated, and accurate procedure for measuring slot and burr. Recently, computer vision and feature extraction are continuously evolving and their popularity is increasing with the help of advanced technology [47,48,49,50]. Table 1 shows a summary of previous image processing techniques used in different machining processes.

There are numerous studies for data extracting related to processing still images using CV technology. This technology offers fast, user-friendly, and accurate solutions for many research areas. Today, most of the studies on obtaining burr formation data and characterization by evaluating SEM images are conducted manually. A screen caliper or an image processing software, such as ImageJ, are common options for manual measurements [32,61,62,63]. However, user abilities and diligence are important parameters to obtain an accurate result from the SEM images. In this field, there are limited studies and resources to extract data from still images using CV technology, which is used for evaluating milling burr formation. Applying this technology to process still SEM images directly without additional steps offers an easy and effective solution while eliminating additional skills required for measuring SEM images. The present study offers a fast and user-friendly solution for measuring slot and burr formation at the micro-scale as well as requiring only one SEM image as input to characterize slot and burr formation with high accuracy. Due to the high speed of the CV technology, the process takes milliseconds for an image and is suitable for evaluating thousands of images in minutes. In addition, the outputs of the solution are suitable for drawing a chart from the obtained data directly. To the best of our knowledge from the literature, the presented method was applied for the first time in the micro-milling process for slot and burr characterization. The accuracy of the proposed method is above 91.26% (minimum accuracy), the processing time for each image is below 0.1 s, and was justified with 7 sample measurements. The approach offered here is an accurate and robust method for precisely measuring slot and burr widths and can be used for slot and burr evaluation in micro-machining applications in the research and industry domains.

## 2. Materials and Methods

### 2.1. Machining Parameters

In this study, IN−718 alloy was chosen as the workpiece material and chemical composition is given in Table 2. Cutting tools with different geometries were used to provide burr formation in different sizes and to determine the success of the image processing method (Table 3). The diameter of each cutting tool was 1 mm. The machining parameters are also given in Table 3. Micro-milling tests were performed under dry conditions. In Table 3, slots A, B, and C belong to the cutting process applied with cutting tools that have the same geometric properties (helix angle 35°, the axial rake angle is −5°, and the number of cutting edges is 3). Slot A is the SEM image captured from the start of the milling process. Slots B and C are the SEM images captured after the cutting lengths of 45 and 135 mm. Other slots correspond to the different tool geometries specified in Table 3. The spindle speed, feed rate, and depth of cut parameters were kept constant for each micro-milling tests. Slots E, F, and G were subjected to a micro-milling process using the same cutting length (10 mm).

**Figure 1:**
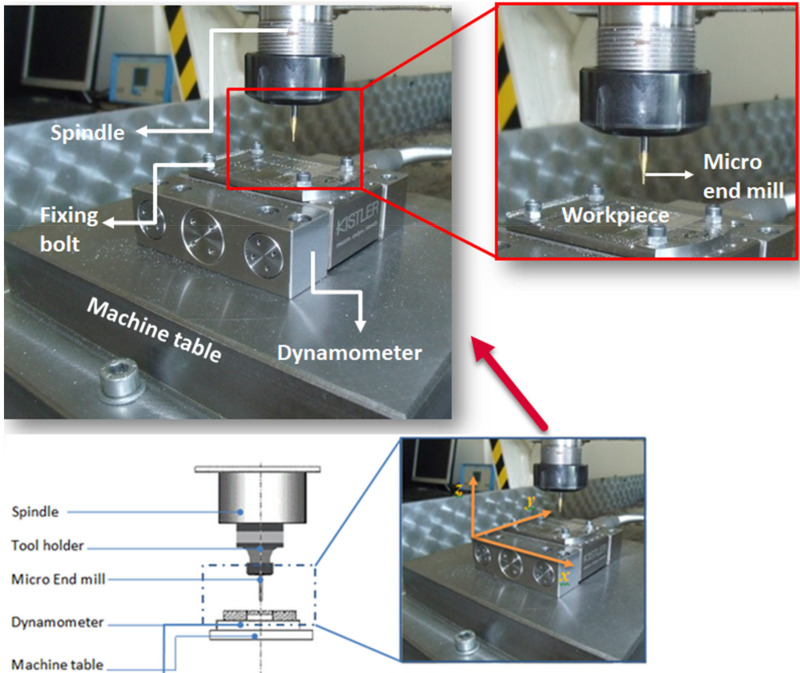
Experimental setup for the micro-milling process.

For the micro-milling tests, a CNC vertical machining center (AKÜ Micro-Machining Laboratory, Afyonkarahisar, Turkey) with a maximum power of 2.2 kW and maximum spindle speed of 24,000 rev/min was used. The experimental setup for the micro-milling process and the details of the micro-end mill, spindle, and workpiece system are given in Figure 1. The workpiece can be connected to the workbench with 4 bolts and the system can be controlled precisely by a computer. The cutting tool is composed of 90% WC and 10% Co. Before the cutting tests, precursory checks were conducted on the axial run out on the spindle and it was determined that the size of the run-out was around 2 μm.

### 2.2. Image Processing Stages

**Figure 2:**
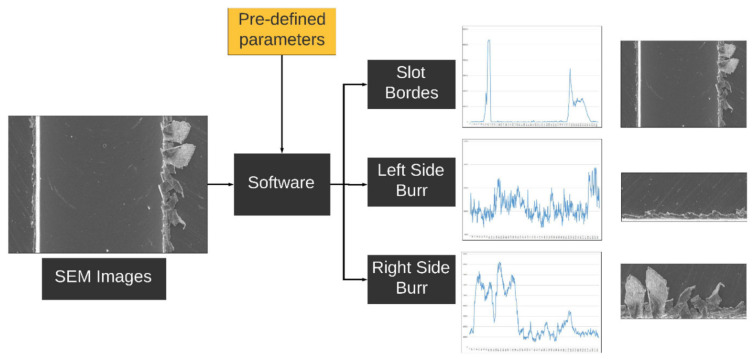
The block diagram of the slot and burr measuring software shows the image input, pre-defined parameters for processing the image, and measurement outputs.

An automated slot and burr measuring software to calculate the borders of the slot and lengths of the burr on a machined workpiece was demonstrated. The software was developed using C++ programming language and the OpenCV library based on computer vision technology. The software algorithm is designed to process SEM images to measure slot and burr using peak points and ROI of the image and a reference scale coefficient in pixels for the predefined filtering parameters. The slot and burr measuring software requires an SEM image, a metric reference coefficient in pixel and predefined threshold parameters for the hue saturation value (HSV), and black-white (BW) transforming operations. The result from the software is the slot and bur parameters using an input SEM image. A block diagram that represents the algorithm of software is presented in Figure 2. Measuring with image processing takes below 0.1 s, which is quite fast when manual measurements on SEM images are considered.

**Figure 3:**
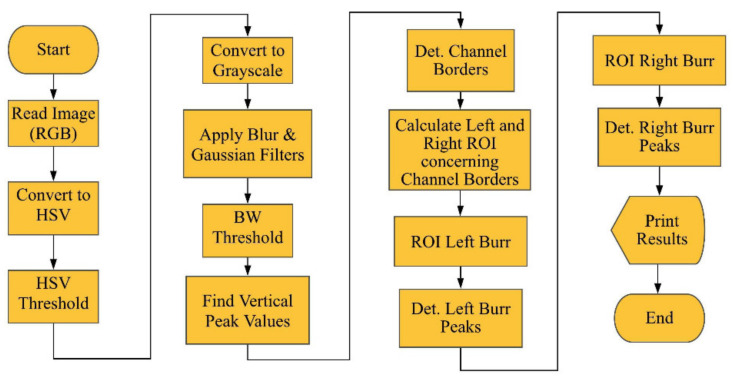
The flowchart of the measuring process.

The measuring process is initiated with an SEM image input. At first, the SEM image obtained by the software was colored. Next, the image is converted to HSV values, and a threshold is applied concerning the pre-defined threshold parameters by the user. The BGR image is masked using the thresholded HSV image. In the next stage, the image is converted to the BW. The Gaussian filter, a blur filter, and a second threshold are applied for minimizing the effect of the background. Then, BGR values are calculated by summing color indents for each column, which is used for finding the vertical peak values. Detected peaks are considered as vertical lines, which represent slot borders. In the next stage, two ROIs are determined according to the slot borders on the up milling side (left side) and down milling side (right side) of the image for calculating the burrs. The ROIs for the up and down milling sides of the images indicate the burrs. These two ROIs are reprocessed using previous steps from the beginning for determining burr lengths using the peak values, but horizontal peak values are considered. A pre-defined coefficient is used to calculate the distances in the metric unit before processing the results. In the result stage, each peak value is used to indicate the determined borders of the slot and length of the burrs for the up and down milling sides of the image. The flowchart of the solution is indicated in Figure 3.

**Figure 4:**
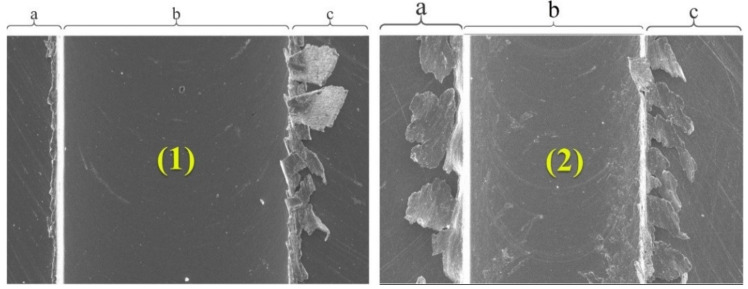
Slot and micro burr segmentation of sample (1) and sample (2): (**a**) micro burrs at the up milling side (left side), (**b**) slots, (**c**) micro burrs at the down milling side (right side).

The slot and burr evaluation process is divided into three main parts by processing the image with three steps. The vertical peak values are determined in the first step and borders are determined with respect to these peaks using the actual image. In the next steps, cropped images are processed for investigating the burr parameters. These images are limited with the slot borders for both burrs of up and down milling sides. The burr parameters related to the peaks are investigated using horizontal peak values from the cropped images (Figure 4).

**Figure 5:**
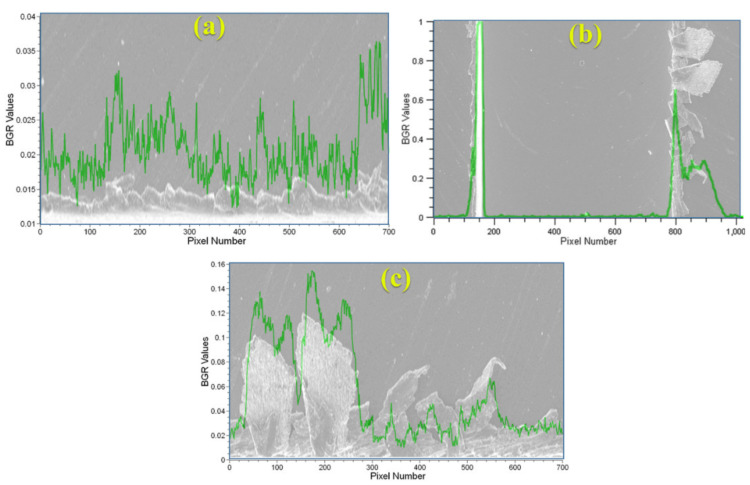
Sample (1): (**a**) automated burr measurement method at the up milling side according to the horizontal peak values, (**b**) automated measurement between the slot borders according to the vertical peak values, and (**c**) the automated burr measurement method at the down milling side according to the horizontal peak values.

**Figure 6:**
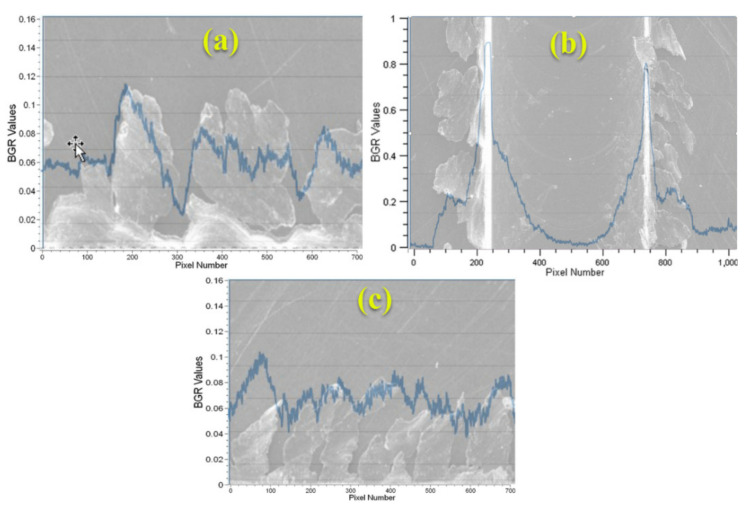
Sample (2): (**a**) automated burr measurement method at the up milling side according to the horizontal peak values, (**b**) automated measurement between the slot borders according to the vertical peak values, and (**c**) the automated burr measurement method at the down milling side according to the horizontal peak values.

The slot determining stage starts after processing the image with respect to the given image processing procedures, vertical peak values are considered for slot length and horizontal peaks for burrs. These peaks are determined by summing the BGR values of each column, which is the vertical and horizontal sum of the pixel indents. The image is divided into two parts for determining the slot borders for the up and down milling sides of the image. The calculated peaks are the maximum values for both the up milling and down milling sides considered as slot borders. The pixel difference of the slot for the *x*-axis gives the length of the slot and the result is converted to metric units by multiplying the difference with a metric coefficient (Figure 5b and Figure 6b).

In the next stage, the ROIs are determined by referencing the detected borders of the slot. Two vertical imaginary lines are drawn on the image to extract the ROIs for the up and down milling sides considering the slot borders. The ROIs mainly include the data about burr parameters. In the ROI extracting step, the maximum X value for the up milling side ROI and the minimum X value for the down milling side ROI was referenced and the limits of the image: the minimum X value and the maximum X value, are used for the up milling and down milling sides ROI, respectively. In the sequel, the two ROIs are generated considering the slot borders and image limits. The ROIs are processed for determining the biggest burr from the peaks and the integral of the chart is used to investigate the burr area. Example results of a processed image for the biggest burr detected from the peaks and burr area for the up milling (Figure 5a and Figure 6a) and down milling (Figure 5c and Figure 6c) sides of the image are shown. In Figure 5 and Figure 6, green and blue colors refer to the vertical peak values for each pixel column that were automatedly calculated by the CV software as indicated in Figure 2. Both figures show that the peak values are increasing with respect to the density of burr formation. The example figure indicates that there is a positive correlation between the determined peak values and burr formation.

In the present study, the mean absolute percentage error (*MAPE*) was used to determine errors for automated measurement with respect to the manual measurements. Therefore, the success rate is obtained by determining errors for each measurement (Equation (1)), where *n* is the number of fitted points, *A_t_* is the actual value, *F_t_* is the forecast value, and ∑ is summation notation:

(1) $$Averaged\;Accuracy\;\left(\%\right)=100-\left[\frac{1}{n}\;\overset{n}{\underset{t=1}{\sum }}\left|\frac{A_{t}-F_{t}}{A_{t}}\right|*100\right]$$

### 2.3. Manual Measurement

**Figure 7:**
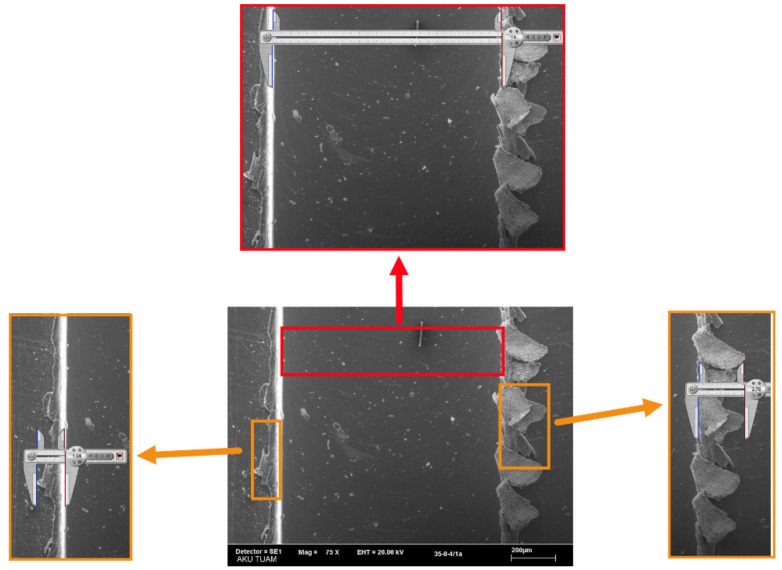
The application of the manual measurement process of slot and burr widths.

After performing the cutting tests, SEM analysis was performed to measure the slot and burr widths manually. The SEM images were taken at different magnifications. The slot and burr widths were measured using Screen Caliper software. Figure 7 shows the application of the manual measurement process of slot and burr widths. The virtual caliper in the screen caliper software was calibrated in terms of pixel-micron conversion, considering the scale of the SEM image. This calibration is valid as long as the SEM image or the scale of the image does not change. By fixing one of the jaws of the virtual caliper and moving the other jaw, both slot and burr widths were measured from several different points.

## 3. Results and Discussions

### 3.1. SEM Images of Slots

**Figure 8:**
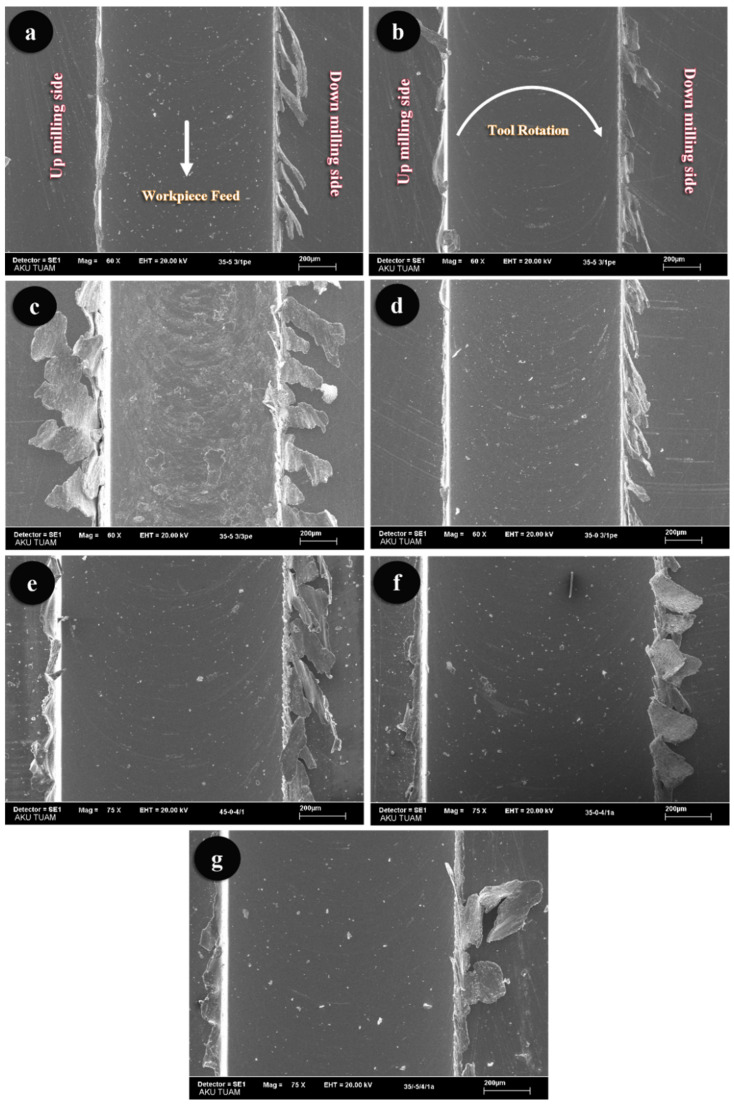
The SEM images of slots; (**a**) Slot-A, (**b**) Slot-B, (**c**) Slot-C, (**d**) Slot-D, (**e**) Slot-E, (**f**) Slot-F, and (**g**) Slot-G.

Figure 8 shows the SEM images of the slots. The SEM images were captured at different magnifications to determine the chances of success rates. The radius of the tool edge and its sharpness decrease as a result of the abrasive wear. The wear of the micro-cutting tool increases due to the cutting length for the same cutting tool and parameters. For all reasons above, the burr widths on the machined slot sides (Figure 8a–c) increased with respect to the cutting length. It was also observed that the burr widths increased due to the effect of the increasing number of cutting edges and helix angles (Figure 8d,e). Additionally, there is no clear effect of the axial rake angle on the burr width. The other prominent finding in Figure 8 is that the burr widths are larger on the down milling sides. However, it is possible to see similar burr widths on the down and up milling sides, which is a possible output of the cutting tool wear. In similar studies, a high correlation is observed for the slot width due to the cutting tool wear [2,31,32,64,65].

### 3.2. Micro Burr Widths for Up Milling and Down Milling Sides

**Figure 9:**
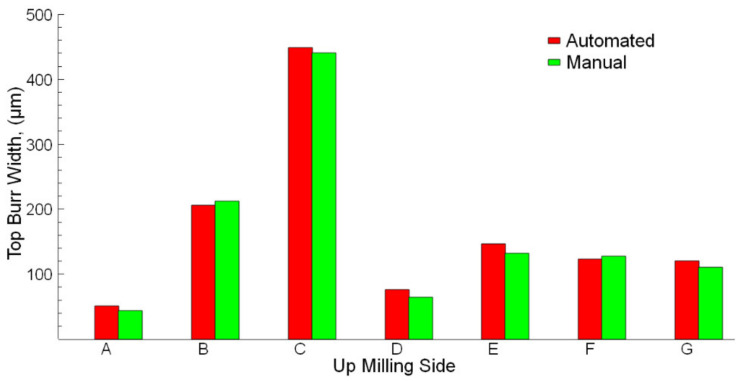
Automated and manual measurements of up milling sides.

**Figure 10:**
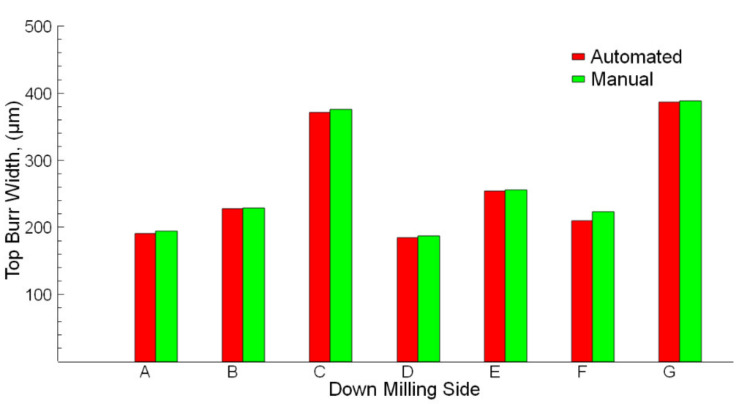
Automated and manual measurements of down milling sides.

The up milling and down milling results are shown in Figure 9 and Figure 10. Measurements of micro burr widths at the up milling and down milling sides were evaluated using both manual and automated methods. Slots A, B, and C belong to the cutting process applied with cutting tools that have the same geometric properties. However, cutting length values for slots A, B, and C are different. Therefore, the effect of cutting length on the burr and slot widths could also be evaluated. In Figure 9 and Figure 10, it is seen that in the micro-milling process of slots A, B, and C, the burr widths measured manually and automatically increase due to the increased cutting lengths. The obtained results from both measurement methods are very close. For slots E, F, and G, the effects of the axial rake angle and number of cutting edges on burr widths are seen. The applied cutting lengths (10 mm) for these slots are minimal in all micro-milling parameters. Therefore, at the beginning of the micro-milling, it is expected that the burr widths are minimal. However, the burr widths for slots E, F, and G are wider than the other slots. The occurrence of this situation is related to the change in the cutting tool geometry used. The axial rake angle and number of cutting edge values for slots E, F, and G are higher than the slots A, B, and C. In a similar study [66], it was predicted similarly to the present study that after the cutting tool used in micro-milling is changed, a cutting tool with higher axial rake angle values is replaced, and the micro-milling process is continued, the burr widths on the up and down milling edges increase.

The results indicate that there is a high correlation between automated and manual measurement results. The obtained results from the experiments show the accuracy of the automated measurements is close to that obtained from the manual measurements. It is observed that the accuracy rates are found to be above 91.3% and 98.2% for up-milling and down-milling, respectively. Mostly, in cutting tests, the down milling side has more burrs compared to the up milling side [32,61,67]. It is understood that the down-milling side burr width is higher than the up-milling side. The same difference is seen from the visual observation on SEM images for the burr widths. In a similar study about the milling process and burr detection, workpieces with different thicknesses and hardness were used. While burrs can be measured very easily after milling thick workpieces, the burrs formed in workpieces less than 2.5 mm thick could not be measured completely by image processing [52]. In the present study, the burrs are in micron sizes and all of the burrs could be measured with over approximately 90% accuracy.

### 3.3. Micro Slot Width

**Figure 11:**
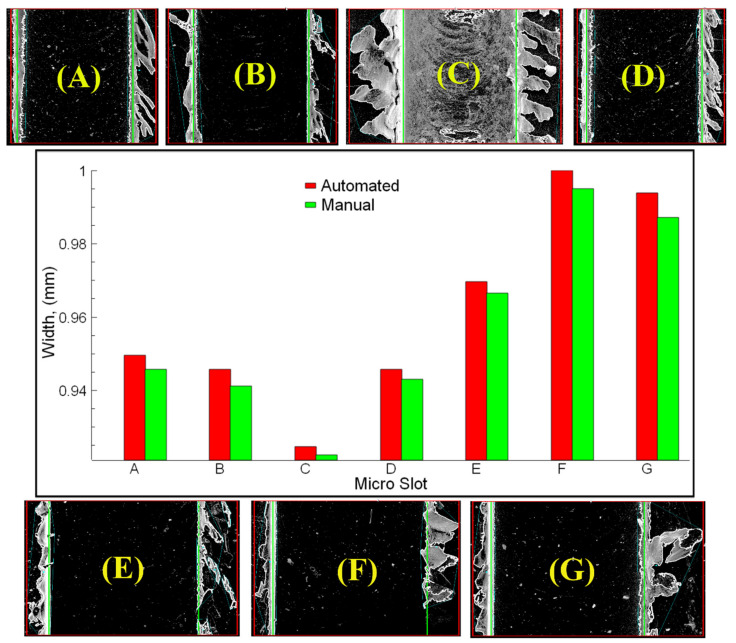
Micro slot widths measured by manual and automated with image process figures; (A) slot-A, (B) slot-B, (C) slot-C, (D) slot-D, (E) slot-E, (F) slot-F, (G) slot-G.

Figure 11 shows the micro slot widths measured by manual and automated with image process figures. Slot borders between green lines (all figures from A to G) indicate the automated measured slot widths. Slot widths are directly dependent on the cutting tool diameter. Therefore, greater wear of the cutting tool is expected due to the increased cutting length. Since the slot width measurement values of E, F, and G in Figure 11 are the values taken from the region close to the starting point, the corresponding slot width values are the closest values to the tool diameter. It is also predicted that in the micro-milling process where the slot width is reduced, there is sign of wear on the cutting tool. In our previous studies [11,31,32,61], it was certainly determined that there is a negative correlation between the cutting tool wear and slot width. In the experiments, cutting tools with approximately a 1000 µm diameter were used with different helix and axial rake angles and number of cutting edges. According to the slot width determining stage results, the accuracy is independent of the tool parameters (indicated in Table 3). The conducted measurements show the accuracy of the automated measurement for determining the slot width is very close to the manual measurement with 99.6% (Figure 11).

### 3.4. Success Rate (%) of Automated and Manual Measurements

**Figure 12:**
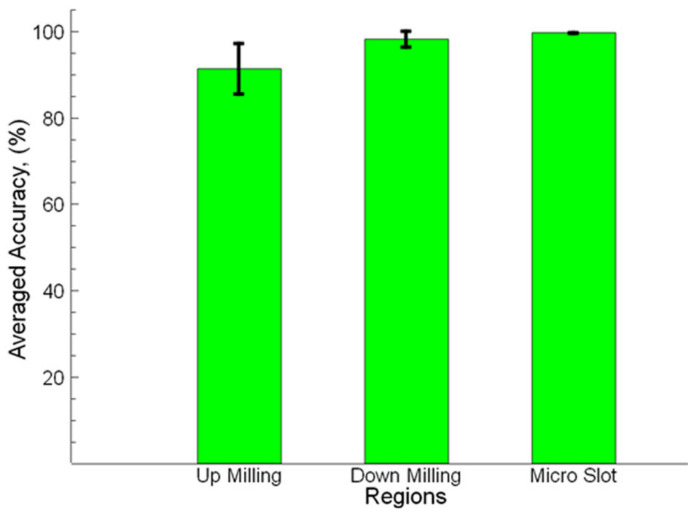
Averaged accuracy for slot and burr widths.

In Figure 12, the averaged accuracy (%), which was calculated according to Equation (1), is shown for the up milling, down milling sides, and slots from seven SEM images. According to the conducted experiments, both the manual and automated measurements addressed accurate results. The measurement results obtained from the up-milling side showed a good correlation with the manual measurements. The result for the up-milling side was 91.26% and a better result was obtained from the down milling side with 98.18%. The slot measurements indicate very close results (99.58%) to the known manual measuring method. The study conducted using the ANN method by Ahn et al. [37] offers a low accuracy of around 80% on the prediction of burr formation in the micromachining process. In another study implemented by Tuiran et al. [39], the burrs resulting from micro-milling were analyzed with the help of an image processing technology, and the accuracy was found to be around 84.46%, which gives the correlation between the spindle speed and burr formation. Medeossi et al. [5] studied burr width evaluation in micro-milling using optical microscopy, and a maximum accuracy was found at 87%. The method used in the present study offers approximately 12%, 8%, and 5% more accuracy compared to the studies implemented by Ahn et al. [37], Tuiran et al. [39], and Medeossi et al. [5], respectively.

In machinability studies, when the measurements are carried out manually, they are completed in a very long time period [68]. In addition, image processing studies could only be carried out based on the results where manual measurement was not performed and machinability tests were carried out on a macro scale [40]. Moreover, there is a low accuracy result of a study in which the measurement of all burrs could not be performed despite the macro-scale milling process [52]. In the present study, in micron-scale, the measurement of the burrs could be carried out precisely and with high accuracy.

## 4. Conclusions

In micro-machining applications, obtaining data from a still SEM image by referencing a scale bar requires multiple image evaluation stages. A screen caliper or an image processing software, such as ImageJ, are common options for manual measurements. However, user abilities and diligence are important parameters to obtain an accurate result from the SEM images. On the other hand, today, CV is a widely used technology in many fields. Applying this technology by processing still SEM images directly without additional steps offers an easy, user-friendly, fast, and accurate solution while eliminating the additional skills required for measuring SEM images. Most of the studies related to image processing techniques require multiple image inputs. The proposed approach can obtain results using a random image input. In the present study, an image processing method was proposed that was compared with a widely used manual burr and slot measurement method. An open-sourced image processing library was used for processing SEM images. The burr and slot measurement software showed a good correlation with the manual measurements. It is a swift, accurate, and time-saving alternative method for slot and burr investigation experiments. The accuracy of the proposed method was above 91%, 98%, and 99% for up milling, down milling, and slot measurements, respectively. Moreover, the accuracy of the results was independent of the SEM images at different magnification and specific tool properties, such as the helix angle, axial rake angle, and number of cutting edges. The proposed image processing method has the potential to investigate the surface quality of micro-machined parts swiftly and accurately.

Using the slot width measurement method, a study can be performed on the comparison of the cutting tool wear rate with the actual measurement results.

## Figures and Tables

**Table 1:** Summary of previous image processing techniques used in different machining processes.

Machining Process	Quality Assessment Scope	Ref.
Milling	Tool wear	[51]
	Burrs	[40]
	Burrs	[52]
	Surface finish	[53]
Drilling	Surface	[54]
	Burrs	[55]
	Burrs	[56]
Turning	Surface roughness	[57]
	Tool wear	[58]
Grinding	Surface finish	[59]
Shaping	Surface finish	[60]

**Table 2:** The chemical composition of IN−718 alloy.

Elements	Cr	Fe	Mo	Nb	Al	Ti	C	Ni
Weight percent wt.%	17–21	16–20	2.8–3.3	4.75–5.5	0.2–0.8	0.65–1.15	0.08 max	Balance

**Table 3:** The properties of cutting tools and machining parameters of the slots.

Slot No	Helix Angle (°)	Axial Rake Angle (°)	Number of Cutting Edges	Spindle Speed (rev/min)	Feed Rate (µm/tooth)	Depth of Cut (µm)	Cutting Length (mm)
Slot A	35	−5	3				10
Slot B	35	−5	3				45
Slot C	35	−5	3				360
Slot D	35	0	3	10,000	3	100	45
Slot E	45	0	4				10
Slot F	35	0	4				10
Slot G	35	−5	4				10

## Data Availability

Experimental data can be obtained by requesting from F.A., A.E. or K.A.

## Nomenclature

SEM	Scanning Electron Microscope
Open CV	Open-sourced Computer Vision
RSM	Response Surface Methodology
ANN	Artificial Neural Network
CV	Computer Vision
AKÜ	Afyon Kocatepe University
HSV	Hue Saturation Value
MAPE	Mean Absolute Percentage Error
BW	Black-White

## References

[1] Kuram E., Ozcelik B. (2017). Optimization of machining parameters during micro-milling of Ti6Al4V titanium alloy and Inconel 718 materials using Taguchi method. Proc. Inst. Mech. Eng. Part B J. Eng. Manuf..

[2] Eraslan D., Balcı A., Çetin B., Uçak N., Çiçek A., Yılmaz O.D., Davut K. (2021). Machinability evaluations of austempered ductile iron and cast steel with similar mechanical properties under eco-friendly milling conditions. J. Mater. Res. Technol..

[3] Giasin K. (2017). Machining Fibre Metal Laminates and Al2024-T3 Aluminium Alloy. Ph.D. Thesis.

[4] Kus A., Isik Y., Cakir M.C., Coşkun S., Özdemir K. (2015). Thermocouple and infrared sensor-based measurement of temperature distribution in metal cutting. Sensors.

[5] Medeossi F., Sorgato M., Bruschi S., Savio E. (2018). Novel method for burrs quantitative evaluation in micro-milling. Precis. Eng..

[6] Li W., Liu M., Ren Y.H., Chen Q. (2019). A high-speed precision micro-spindle use for mechanical micro-machining. Int. J. Adv. Manuf. Technol..

[7] Akkoyun F., Ozcelik A. (2020). A simple approach for controlling an open-source syringe pump. Eur. Mech. Sci..

[8] Jung W.C., Heo Y.M., Yoon G.S., Shin K.H., Chang S.H., Kim G.H., Cho M.W. (2007). Micro machining of injection mold inserts for fluidic channel of polymeric biochips. Sensors.

[9] Lin Y.S., Yang C.H., Wang C.Y., Chang F.R., Huang K.S., Hsieh W.C. (2012). An aluminum microfluidic chip fabrication using a convenient micromilling process for fluorescent poly(DL-lactide-co-glycolide) microparticle generation. Sensors.

[10] Lauro C.H., Brand L.C., Panzera T.H., Davim J.P. (2015). Surface integrity in the micromachining: A review. Rev. Adv. Mater. Sci..

[11] Aslantas K., Alatrushi L.K.H. (2020). Experimental study on the effect of cutting tool geometry in micro-milling of Inconel 718. Arab. J. Sci. Eng..

[12] Bhuvanesh Kumar M., Sathiya P., Parameshwaran R. (2020). Parameters optimization for end milling of Al7075–ZrO2–C metal matrix composites using GRA and ANOVA. Trans. Indian Inst. Met..

[13] Sun Q., Cheng X., Zhao G., Yang X., Zheng G. (2019). Experimental study of micromilling burrs of 304 stainless steel. Int. J. Adv. Manuf. Technol..

[14] Kuntoğlu M., Aslan A., Pimenov D.Y., Usca Ü.A., Salur E., Gupta M.K., Mikolajczyk T., Giasin K., Kapłonek W., Sharma S. (2021). A review of indirect tool condition monitoring systems and decision-making methods in turning: Critical analysis and trends. Sensors.

[15] Perçin M., Aslantas K., Ucun I., Kaynak Y., Çicek A. (2016). Micro-drilling of Ti−6Al−4V alloy: The effects of cooling/lubricating. Precis. Eng..

[16] Kumar M.B., Parameshwaran R., Deepandurai K., Senthil S.M. (2020). Influence of milling parameters on surface roughness of Al–SiC–B4C composites. Trans. Indian Inst. Met..

[17] Li M., Huang M., Jiang X., Kuo C.L., Yang X. (2018). Study on burr occurrence and surface integrity during slot milling of multidirectional and plain woven CFRPs. Int. J. Adv. Manuf. Technol..

[18] Wojciechowski S., Matuszak M., Powałka B., Madajewski M., Maruda R.W., Królczyk G.M. (2019). Prediction of cutting forces during micro end milling considering chip thickness accumulation. Int. J. Mach. Tools Manuf..

[19] Yadav A.K., Kumar M., Bajpai V., Singh N.K., Singh R.K. (2017). FE modeling of burr size in high- speed micro-milling of Ti6Al4V. Precis. Eng..

[20] Khanghah S.P., Boozarpoor M., Lotfi M., Teimouri R. (2015). Optimization of micro-milling parameters regarding burr size minimization via RSM and simulated annealing algorithm. Trans. Indian Inst. Met..

[21] Jeong H., Ha J., Hwang J., Lee H., Kim D., Kim N. (2014). A study on the shearing process and the burr formation of zircaloy−4 sheet by using GTN model. Int. J. Precis. Eng. Manuf..

[22] Yoon H.S., Wu R., Lee T.M., Ahn S.H. (2011). Geometric optimization of micro drills using Taguchi methods and response surface methodology. Int. J. Precis. Eng. Manuf..

[23] Aamir M., Tu S., Tolouei-Rad M., Giasin K., Vafadar A. (2020). Optimization and modeling of process parameters in multi-hole simultaneous drilling using taguchi method and fuzzy logic approach. Materials.

[24] Aamir M., Tolouei-Rad M., Vafadar A., Raja M.N.A., Giasin K. (2020). Performance analysis of multi-spindle drilling of Al2024 with TiN and TiCN coated drills using experimental and artificial neural networks technique. Appl. Sci..

[25] Liu C., Shi B., Zhou J., Tang C. (2011). Quantification and characterization of microporosity by image processing, geometric measurement and statistical methods: Application on SEM images of clay materials. Appl. Clay Sci..

[26] Abdallah R., Soo S.L., Hood R. (2021). The influence of cut direction and process parameters in wire electrical discharge machining of carbon fibre–reinforced plastic composites. Int. J. Adv. Manuf. Technol..

[27] Giasin K., Barouni A., Dhakal H.N., Featherson C., Redouane Z., Morkavuk S., Koklu U. (2021). Microstructural investigation and hole quality evaluation in S2/FM94 glass-fibre composites under dry and cryogenic conditions. J. Reinf. Plast. Compos..

[28] Yin Q., Liu Z., Wang B., Song Q., Cai Y. (2020). Recent progress of machinability and surface integrity for mechanical machining Inconel 718: A review. Int. J. Adv. Manuf. Technol..

[29] Aslantas K., Ekici E., Çiçek A. (2018). Optimization of process parameters for micro milling of Ti−6Al−4V alloy using Taguchi-based gray relational analysis. Measurement.

[30] Krolczyk G.M., Maruda R.W., Krolczyk J.B., Nieslony P., Wojciechowski S., Legutko S. (2018). Parametric and nonparametric description of the surface topography in the dry and MQCL cutting conditions. Meas. J. Int. Meas. Confed..

[31] Erçetin A., Aslantaş K., Perçin M. (2018). Micro milling of tungsten-copper composite materials produced through powder metallurgy method: Effect of composition and sintering temperature. J. Fac. Eng. Archit. Gazi Univ..

[32] Erçetin A., Aslantas K., Özgün Ö. (2020). Micro-end milling of biomedical TZ54 magnesium alloy produced through powder metallurgy. Mach. Sci. Technol..

[33] Koklu U., Morkavuk S., Featherston C., Haddad M., Sanders D., Aamir M., Pimenov D.Y., Giasin K. (2021). The effect of cryogenic machining of S2 glass fibre composite on the hole form and dimensional tolerances. Int. J. Adv. Manuf. Technol..

[34] Ercetin A. (2021). Application of the hot press method to produce new Mg alloys: Characterization, mechanical properties, and effect of Al addition. J. Mater. Eng. Perform..

[35] Varatharajulu M., Duraiselvam M., Arun Kumar K., Gabrial Kanniyan C., Sathiyamurthy R. (2021). Experimental investigation of the effect of independent parameters in the face milling of aluminum 6082 alloy. Trans. Indian Inst. Met..

[36] Kapłonek W., Nadolny K., Krolczyk G., Królczyk G.M. (2016). The use of focus-variation microscopy for the assessment of active surfaces of a new generation of coated abrasive tools. Meas. Sci. Rev..

[37] Ahn Y., Lee S.H. (2017). Classification and prediction of burr formation in micro drilling of ductile metals. Int. J. Prod. Res..

[38] Giasin K., Ayvar-Soberanis S., French T., Phadnis V. (2017). 3D finite element modelling of cutting forces in drilling fibre metal laminates and experimental hole quality analysis. Appl. Compos. Mater..

[39] Tuirán R., Oñate J., Romero N. (2018). Analysis of burr formation by image processing in micro-milling of Ti. Contemp. Eng. Sci..

[40] Sharan R.V., Onwubolu G.C. (2011). Measurement of end-milling burr using image processing techniques. Proc. Inst. Mech. Eng. Part B J. Eng. Manuf..

[41] Mikołajczyk T., Nowicki K., Bustillo A., Pimenov D.Y. (2018). Predicting tool life in turning operations using neural networks and image processing. Mech. Syst. Signal Process..

[42] Mikołajczyk T., Nowicki K., Kłodowski A., Pimenov D.Y. (2017). Neural network approach for automatic image analysis of cutting edge wear. Mech. Syst. Signal Process..

[43] Schneider C.A., Rasband W.S., Eliceiri K.W. (2012). NIH image to imageJ: 25 years of image analysis. Nat. Methods.

[44] Aurich J.C., Dornfeld D., Arrazola P.J., Franke V., Leitz L., Min S. (2009). Burrs-Analysis, control and removal. CIRP Ann. Manuf. Technol..

[45] Ko S.L., Park S.W. (2006). Development of an effective measurement system for burr geometry. Proc. Inst. Mech. Eng. Part B J. Eng. Manuf..

[46] Toropov A. (2007). An Effective Visualization and Analysis Method for Edge Measurement. Computational Science and Its Applications.

[47] Sardoğan M., Özen Y., Tuncer A. (2020). Detection of apple leaf diseases using faster R-CNN. Düzce Univ. J. Sci. Technol..

[48] Hiçdurmaz A., Tuncer A. (2020). Real-time obstacle avoidance based on floor detection for mobile robots. Sak. Univ. J. Sci..

[49] Xi T., Benincá I.M., Kehne S., Fey M., Brecher C. (2021). Tool wear monitoring in roughing and finishing processes based on machine internal data. Int. J. Adv. Manuf. Technol..

[50] Li J., Lu J., Chen C., Ma J., Liao X. (2021). Tool wear state prediction based on feature-based transfer learning. Int. J. Adv. Manuf. Technol..

[51] Dai Y., Zhu K. (2018). A machine vision system for micro-milling tool condition monitoring. Precis. Eng..

[52] Chen X., Shi G., Xi C., Zhong L., Wei X., Zhang K. (2019). Design of Burr Detection Based on Image Processing. Journal of Physics: Conference Series.

[53] Kumar R., Kulashekar P., Dhanasekar B., Ramamoorthy B. (2005). Application of digital image magnification for surface roughness evaluation using machine vision. Int. J. Mach. Tools Manuf..

[54] Liu W., Zheng X., Liu S., Jia Z. (2012). A roughness measurement method based on genetic algorithm and neural network for microheterogeneous surface in deep-hole parts. J. Circuits Syst. Comput..

[55] Yoon H.S., Chung S.C. (2004). Vision inspection of micro-drilling processes on the machine tool. Trans. N. Am. Manuf. Res. Inst. SME.

[56] Nakao Y., Watanabe Y. (2006). Measurements and evaluations of drilling burr profile. Proc. Inst. Mech. Eng. Part B J. Eng. Manuf..

[57] Sarma P.M.M.S., Karunamoorthy L., Palanikumar K. (2009). Surface roughness parameters evaluation in machining GFRP composites by PCD tool using digital image processing. J. Reinf. Plast. Compos..

[58] Dutta S., Datta A., Chakladar N.D., Pal S.K., Mukhopadhyay S., Sen R. (2012). Detection of tool condition from the turned surface images using an accurate grey level co-occurrence technique. Precis. Eng..

[59] Vesselenyi T., Dzitac I., Dzitac S., Vaida V. (2008). Surface roughness image analysis using quasi-fractal characteristics and fuzzy clustering methods. Int. J. Comput. Commun. Control.

[60] Priya P., Ramamoorthy B. (2010). Machine Vision for Surface Roughness Assessment of Inclined Components. Key Engineering Materials.

[61] Erçetin A., Aslantaş K. (2016). The effect of different cutting parameters on cutting force, tool wear and burr formation in micro milling WCu composite material fabricated via powder metallurgy. Turk. J. Nat. Sci..

[62] García-Ordás M.T., Alegre-Gutiérrez E., Alaiz-Rodríguez R., González-Castro V. (2018). Tool wear monitoring using an online, automatic and low cost system based on local texture. Mech. Syst. Signal Process..

[63] D’Addona D.M., Ullah A.M.M.S., Matarazzo D. (2017). Tool-wear prediction and pattern-recognition using artificial neural network and DNA-based computing. J. Intell. Manuf..

[64] Uçak N., Aslantas K., Çiçek A. (2020). The effects of Al2O3 coating on serrated chip geometry and adiabatic shear banding in orthogonal cutting of AISI 316L stainless steel. J. Mater. Res. Technol..

[65] Gao Q., Guo G.Y., Cai M. (2021). Wear mechanism and experimental study of a tool used for micro-milling single-crystal nickel-based superalloys. Int. J. Adv. Manuf. Technol..

[66] Afazov S.M., Zdebski D., Ratchev S.M., Segal J., Liu S. (2013). Effects of micro-milling conditions on the cutting forces and process stability. J. Mater. Process. Technol..

[67] Kuntoğlu M., Acar O., Gupta M.K., Sağlam H., Sarikaya M., Giasin K., Pimenov D.Y. (2021). Parametric optimization for cutting forces and material removal rate in the turning of AISI 5140. Machines.

[68] Erçetin A., Usca Ü.A. (2016). An experimental investigation of effect of turning AISI 1040 steel at low cutting speed on tool wear and surface roughness. Turk. J. Nat. Sci..

